# Locally advanced and metastatic esophageal squamous cell carcinoma in Morocco: from diagnosis to treatment

**DOI:** 10.3332/ecancer.2025.1882

**Published:** 2025-04-01

**Authors:** Anass Baladi, Hassan Abdelilah Tafenzi, Fatim-Zahra Megzar, Ibrahima Kalil Cisse, Othmane Zouiten, Leila Afani, Ismail Essaadi, Mohammed El Fadli, Rhizlane Belbaraka

**Affiliations:** 1 Department of Medical Oncology, Mohammed VI University Hospital of Marrakech, Marrakesh 40000, Morocco; 2 Biosciences and Health Laboratory, Faculty of Medicine and Pharmacy, Cadi Ayyad University, Marrakech 40000, Morocco; 3 Medical Oncology Department, Avicenna Military Hospital of Marrakech, Marrakech 40000, Morocco

**Keywords:** esophageal cancer, esophageal squamous cell carcinoma, prognostic factors

## Abstract

**Background:**

Esophageal squamous cell carcinoma (ESCC) is the most common subtype of esophageal cancer (EC) worldwide, with significant geographic variability in its incidence and outcomes. This study aims to analyse the characteristics of Moroccan ESCC patients, identify independent prognostic factors for mortality and assess access to surgery, chemotherapy, radiotherapy (RT) and targeted therapies.

**Methods:**

This retrospective study analysed data from the Marrakesh-Safi regional cancer registry. Between 2014 and 2019, a total of 78 patients were histologically confirmed to have locally advanced or metastatic ESCC. Demographic, clinical and treatment data were evaluated to determine prognostic factors for overall survival (OS) using Kaplan–Meier and multivariate Cox regression analyses.

**Results:**

The median age was 56 years (IQR: 48–66), with a slight female predominance in stage III (59%). Dysphagia was the most frequent symptom (92%), and the thoracic esophagus was the most common tumour site (53%). Performance status was significantly worse in stage IV (31% with PS 4, *p *< 0.001). Chemotherapy was administered to 72% of patients, with cisplatin being the most used drug. RT was more common in stage III (57% versus 33%, *p* = 0.035), while surgery was rare (2 cases). Multivariate analysis identified performance status as a key prognostic factor (HR = 27.2, *p* = 0.015), while RT significantly reduced mortality risk (HR = 0.07, *p* = 0.038). Stage III patients had a median OS of 46 months, with 1- and 3-year OS rates of 84% and 78%, respectively. In contrast, stage IV patients had a median OS of 8.6 months, with 1-year and 3-year OS rates of 34% and 22%, respectively.

**Conclusion:**

Patients with locally advanced or metastatic EC face poor survival outcomes. RT and performance status are key factors that significantly influence prognosis. These findings underscore the urgent need for early detection, enhanced access to multimodal treatments and improved healthcare infrastructure to improve survival outcomes.

## Introduction

Esophageal cancer (EC) remains a significant global health concern, with varying trends in its incidence. According to a comprehensive analysis reported by Mazidimoradi *et al* [1], there were over 600,000 new cases of EC in 2020, with esophageal squamous cell carcinoma (ESCC) being the predominant subtype, especially in Eastern Asia, South Central Asia and Sub-Saharan Africa. Overall, the age-standardised rates for EC, including both incidence and mortality, have been declining globally since 1990, with variations across different regions and countries [2]. These statistics underscore the importance of continued research and targeted prevention strategies to address the burden of EC on a global scale.

In North Africa, and particularly in Morocco, reliable epidemiological data on EC remains scarce. However, the World Health Organisation highlights the growing burden of cancer in Morocco, where EC in Morocco ranks 23rd in incidence among all cancer types and 17th in terms of cancer-related deaths. The lifetime risk of death from EC is estimated to be around 1.1% [3]. According to the latest cancer statistics, EC, particularly ESCC, is linked to various common risk factors globally. Studies have identified key risk factors such as tobacco and alcohol use, polycyclic aromatic hydrocarbon exposure, hot food and beverage consumption, poor oral health and a diet low in fruits and vegetables [4–6]. Understanding these diverse risk factors is crucial for developing targeted prevention strategies and interventions to reduce the global burden of EC effectively [7]. For instance, although the 5-year survival rate for distant ESCC is barely 5%, it is 46.4% for localised ESCC [8]. Its poor prognosis is mainly based on the lack of early detection of precancerous lesions due to the late consultation of patients with the first symptom. Unfortunately, EC is most often discovered late, and most often, at the time of diagnosis, the cancer is already advanced and unresectable, given the anatomical location of the esophagus and its contiguity with vital organs [9]. To manage ESCC patients appropriately and increase their overall survival (OS) probability, an early and precise identification of the disease is crucial. EC is frequently found after it is already advanced, necessitating very invasive therapies such as surgical resection and chemoradiotherapy [10].

Due to a lack of information regarding North African ESCC, in this study, we brought to light the actual situation concerning patients with ESCC from diagnosis to treatment, all by detailing features, the independent prognostic factors related to OS and the accessibility of treatments from surgery, chemotherapy, radiotherapy (RT) and targeted therapies, to provide insights and the most recent data.

## Materials and methods

Patients diagnosed with ESCC between 2014 and 2019 were identified from the medical oncology department of Mohammed VI University Hospital in Marrakech, based on their stage at diagnosis. The date of occurrence was considered the day of the biopsy. Follow-up data for each patient were compiled using the most recent medical records, including clinical examinations and recent computed tomography (CT) assessments. In cases where patients were lost to follow-up, they were contacted using their phone numbers. Only patients who met the following criteria were eligible for this study: those histologically diagnosed with ESCC as a primary malignancy confirmed either by biopsy between 2014 and 2019 and those who had clinically locally advanced or metastatic disease. Patients were excluded if they had documented clinical T0 (no evidence of a primary tumour), Tis (high-grade dysplasia) or if they were diagnosed at early stages (I-IIb). RT and chemotherapy, with or without surgery, are recognised as part of a bi- or tri-modal curative-intention approach for EC, in line with global standards. Survival was defined as the period from the patient’s enrollment date to the date of death. For patients lost to follow-up, we made efforts to contact families to ascertain the date of death, thereby ensuring the accuracy of our survival data. Data for patients who were still living and had not experienced a relapse were censored as of the last known follow-up appointment date.

Patient characteristics for ESCC were systematically collected, including sex (male, female), age at diagnosis and smoking status (yes, no), as well as initial symptoms (dysphagia, dyspnea, vomiting, epigastric pain and general condition impairment). Histopathological data comprised the anatomical location, tumour differentiation and macroscopic tumour features (ulcerative-budding and stenosing, stenosing, ulcer-infiltrative, infiltrative, ulcer-budding and non-specific appearance). Tumour invasion was assessed using CT scans by evaluating lymphadenopathy in regions such as the subcarinal, mediastinal, jugular-carotid and perigastric nodes. The scans also identified tumour contact with adjacent structures, including the descending aorta and the para-laryngeal region. The PS was evaluated using the Eastern Cooperative Oncology Group Performance Status (ECOG PS), and clinical T, N and M categories and cancer stage at diagnosis were determined according to the 7th edition of the American Joint Committee on Cancer (AJCC). Metastatic sites (lung, liver, bone and adrenal) were also recorded at diagnosis. In cases where specific T, N and M details were unavailable, the overall stage according to the 7th edition of AJCC, as documented in the medical records, was used, reflecting the clinician’s synthesis of available diagnostic data. We documented the administration of various treatments for ESCC, specifying whether patients received surgery, RT or chemotherapy.

RT is crucial for treating ESCC. Neoadjuvant chemoradiotherapy improves survival and surgical outcomes. For patients who cannot undergo surgery, definitive RT, with or without chemotherapy, is an option. Palliative RT effectively relieves dysphagia and pain. For chemotherapy, we detailed the specific regimens used, which included 5-fluorouracil (5-FU), Folfox (a combination of Oxaliplatin, 5-FU and Leucovorin), Xelox (a combination of Capecitabine and Oxaliplatin), Xeloda (Capecitabine), Cisplatin and Docetaxel. The treatment administration was recorded as either ‘Yes’ if the patient received the treatment or ‘No’ if they did not.

Categorical variables were expressed as percentages, while continuous variables such as age were expressed as medians and quartiles. Cox proportional hazard regression analyses were used to identify the independent factors related to OS in patients diagnosed with ESCC. The selection of the most appropriate statistical test depends on the specific characteristics of the variable under consideration. Tests such as the Wilcoxon rank-sum test, Pearson’s chi-squared test and Fisher’s exact test are chosen based on the unique requirements of the data. Additionally, the Kaplan–Meier method was used to estimate all time-to-event distributions based on prognostic variables selected from the multivariate Cox proportional hazard regression analysis and compared by the log-rank test. A *p*-value of less than 0.05 was considered statistically significant. The ‘survival’ and ‘survminer’ packages in R software were used for all statistical analyses.

## Results

### Description

Over the 5 years from 2014 to 2019, the study included 78 patients diagnosed with ESCC, divided into stage III (*n* = 42) and stage IV (*n* = 36). The population in our study represents a regional subset of ESCC patients treated specifically at the Mohammed VI University Hospital in the Marrakesh-Safi region of Morocco. The median follow-up period was approximately 8.5 months (0 to 48.9 months). The demographic, clinical, pathological and therapeutic characteristics of the patients with both locally advanced and metastatic ESCC are summarised in Table 1.

The median age at diagnosis of the patients was 56 years (IQR: 48–66), with no significant difference between stages. Females were slightly predominant (59%) in stage III (*p* = 0.14). Dysphagia was the most common symptom, affecting 92% of patients. The most frequent tumour location was the thoracic esophagus, observed in 40% of patients and the predominant macroscopic appearance was stenosing (48%). PS was significantly worse in stage IV, with 31% of patients having a PS of 4 (*p* < 0.001), indicating a marked deterioration in physical condition at advanced stages of the disease. Among the observed metastases, lung metastases were the most common (24%), followed by liver (9%), bone (7.7%) and adrenal metastases (2.6%). No brain metastases or other distant metastatic sites outside the recorded categories were observed in our cohort.

Among the 78 patients in the study, 22 (28%) did not receive chemotherapy, comprising 9 patients with stage III and 13 with stage IV. Of the 56 patients (72%) who received chemotherapy, 13 with stage III underwent neoadjuvant chemotherapy, 10 received concurrent chemotherapy and 10 received palliative chemotherapy. In contrast, 23 patients with stage IV were treated with palliative chemotherapy. No significant difference in the overall administration of chemotherapy was observed between stage III and stage IV patients (*p* = 0.15). The most frequently used chemotherapy agents were cisplatin and 5FU, with Xeloda and Xelox used less commonly. RT was more commonly administered to patients in stage III, with 57% receiving localised RT, compared to 33% of stage IV patients who received metastatic RT. Stage III patients received a median of 15 RT sessions with 30 total fractions, while stage IV patients received a median of 10 sessions with 20 total fractions. Surgical intervention was rare, with only 2 out of 78 patients undergoing surgery – one diagnosed at stage III and one at stage IV.

### Survival analysis

Multivariate Cox regression analysis identified independent prognostic factors associated with survival. PS emerged as a key factor, with patients having a PS of 3 showing a significantly increased mortality risk (HR = 27.2, 95% CI: 1.87–394, *p* = 0.015), making PS a strong independent predictor of poor prognosis. Additionally, RT proved to be an important protective factor, significantly reducing the risk of mortality (HR = 0.07, 95% CI: 0.01–0.86, *p* = 0.038). In contrast, chemotherapy was not significantly associated with improved survival after adjusting for other variables (HR = 0.48, 95% CI: 0.07–3.36, *p* = 0.5) (Table 2).

**Figure 1.**
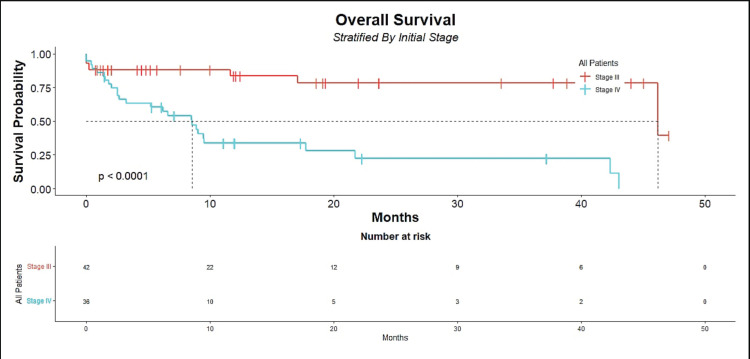
OS in patients diagnosed with ESCC as the primary malignancy stratified by stage at diagnosis.

For patients with stage III, the median OS was 46 months (95% CI, 46 to not estimable). The 1-year OS rate was 84% (95% CI, 72% to 97%), and the 3-year OS rate was 78% (95% CI, 64% to 95%). In contrast, the median OS for patients with stage IV was 8.6 months (95% CI, 3.3 to 22). The 1-year OS rate was 34% (95% CI, 21% to 55%), and the 3-year OS rate was 22% (95% CI, 11% to 48%) (Figure 1).

## Discussion

ESCC is the predominant histological subtype of EC in various regions, including Morocco and Africa. Research indicates that ESCC remains the primary subtype of EC globally, especially in regions like Eastern Asia, South Central Asia and Sub-Saharan Africa [11]. Additionally, the African ESCC corridor, spanning from Ethiopia to South Africa, highlights the disproportionately high incidence and mortality rates of EC in this region, emphasising the prevalence of ESCC [12]. Therefore, both global and regional data support the emergence of ESCC as the predominant histological subtype of EC in Morocco and Africa. This subtype is marked by high incidence rates and a generally poor prognosis worldwide [13]. Concerning Africa, currently, no studies have been reported to compare and analyse the morbidity and prognosis between the Moroccan population and other African, European, American and Asian regions [14].

Delays in diagnosis remain a significant barrier to improving outcomes for patients with ESCC in Morocco. The average diagnostic interval in Morocco surpasses international recommendations, with patients waiting approximately 52 days from symptom onset to diagnosis [14]. This delay is driven by several interrelated factors, including socioeconomic challenges, cultural and geographical barriers and systemic healthcare limitations. Socioeconomic challenges play a critical role in impeding timely diagnosis and treatment. Financial constraints and limited access to healthcare facilities disproportionately affect patients from lower-income groups, particularly those covered by the Régime d’Assistance Médicale (RAMED) system RAMED system, which is Morocco’s medical assistance program designed to provide healthcare access to economically disadvantaged populations. Economic barriers significantly hinder patients’ ability to seek and receive adequate oncology care [15]. Reliance on traditional, complementary and integrative medicine can lead to critical delays, as patients may prioritise these remedies over seeking immediate medical care [16].

In our study, the independent prognostic factors associated with survival in ESCC include the effects of RT and PS. RT has emerged as a crucial protective factor in reducing mortality risk in patients with ESCC. Studies indicate that both postoperative radiotherapy (PORT) and definitive chemoradiotherapy (dCRT) significantly enhance survival outcomes, particularly when combined with chemotherapy. PORT has been shown to improve local-regional recurrence-free survival and OS in patients undergoing surgical resection [17]. dCRT, particularly with advanced techniques like intensity-modulated radiotherapy, has demonstrated improved 5-year OS rates of 30.9% in non-surgically resectable cases [18]. Neoadjuvant chemoradiotherapy has been associated with improved surgical outcomes and survival compared to surgery alone, highlighting its role in managing locally advanced ESCC [19]. These findings suggest a more aggressive treatment approach in patients with locally advanced disease (stage III), where RT, often combined with chemotherapy, plays a central role. Palliative RT offers significant survival benefits for patients with EC, particularly in managing dysphagia, a common symptom affecting quality of life. Studies indicate that external beam radiotherapy can alleviate dysphagia in approximately 82.45% of patients, with a notable decrease in dysphagia scores post-treatment [20]. Furthermore, palliative RT has been associated with improved OS and cancer-specific survival in metastatic cases, with median OS extending significantly for those receiving radiation compared to non-receivers [21, 22]. While RT significantly enhances survival rates in ESCC, concerns regarding treatment-related toxicities persist, necessitating careful patient selection and treatment planning to optimise outcomes [17].

Research indicates that PS significantly predicts survival outcomes, influencing treatment decisions and patient stratification. PS is a critical measure of a patient’s overall health, and scales like ECOG and Karnofsky are commonly used. A study highlighted that PS significantly impacts survival, with better PS correlating with improved outcomes in various cancers, including ESCC [23]. Specifically, in patients receiving palliative treatment, a better PS was associated with 2.56 times greater odds of survival [24]. In our study, patients with PS III and IV exhibited significantly worse prognoses due to their inability to tolerate standard therapies. All patients with PS III or IV received only palliative supportive care, underscoring a critical gap in addressing the needs of this subgroup.

These findings emphasise the urgent need to develop tailored interventions, such as dose-reduced or symptom-targeted therapies, to better meet the unique needs of these patients [25, 26]. Early integration of supportive and palliative care may also improve quality of life and optimise outcomes [27]. Exploring these approaches in the Moroccan context could help address this unmet need. Increased awareness of EC risk factors has been associated with shorter intervals between symptom onset and diagnosis. This underscores the importance of community-based education programs to inform individuals about early symptoms and the need for prompt medical consultation [28, 29]. Addressing systemic barriers such as financial constraints, limited health literacy and inadequate healthcare infrastructure is crucial for reducing diagnostic delays [30, 31] Cost-effective screening methods tailored to local contexts could facilitate earlier detection, while international collaborations may help develop effective health policies and enhance cancer care capacity in low- and middle-income countries (LMICs) [31, 32]. Efforts in other LMICs, such as Kenya’s mobile diagnostic units, demonstrate the potential for community-level solutions to improve early detection [33]. Addressing these priorities is essential for improving survival outcomes and quality of life for ESCC patients in Morocco. Future research should focus on multicenter studies to validate these findings and assess the long-term impact of proposed interventions.

In our study, chemotherapy did not significantly improve survival, which may be attributed to several factors. Advanced-stage disease and poor PS significantly hinder patients’ ability to tolerate standard chemotherapy regimens, as evidenced by studies indicating that treatment delays and toxicity can diminish efficacy and OS in advanced cancer patients [34, 35]. Additionally, the concept of ‘time toxicity’ highlights the burden of healthcare interactions, which can detract from quality of life and potentially lead to treatment interruptions [36]. Furthermore, patients often prioritise quality of life over progression-free survival when faced with treatments that do not offer OS benefits, indicating a complex decision-making landscape [37].

### Study limitations

The main limitations of this study include the potential for recall bias due to its retrospective design, as well as missing data for certain parameters, such as clinical T category and tobacco use. Additionally, the study was conducted at a single institution, which may not fully represent the clinical, demographic and socioeconomic diversity of the broader Moroccan population. The relatively small sample size of 78 patients further limits the generalisability of the findings, highlighting the need for larger, multicenter studies to confirm these results. The imbalance in PS scores may have introduced bias, as patients with better PS are more likely to tolerate aggressive treatments, potentially inflating survival outcomes. The high proportion of censored patients, especially in stage III, reduced the number at risk at later time points but did not affect the accuracy of the median survival, calculated using observed events with the Kaplan–Meier method. Furthermore, key risk factors, including alcohol consumption, family history, dietary habits and hot beverage consumption, were not addressed in this study due to the limitations of retrospective data and the need for prospective data collection.

## Conclusion

This retrospective study underscores the poor survival outcomes and identifies key prognostic factors influencing survival in ESCC patients treated at the Mohammed VI University Hospital in the Marrakesh-Safi region of Morocco. PS is a strong independent prognostic factor for survival in ESCC, with poorer PS associated with significantly higher mortality. Early detection of EC is crucial for improving survival chances, emphasising the importance of improving access to timely diagnostic and treatment services, investing in healthcare infrastructure and enhancing oncologist training.

## List of abbreviations

AJCC, American Joint Committee on Cancer; dCRT, definitive Chemo-RadioTherapy; ECOG PS, Eastern Cooperative Oncology Group Performance Status; ESCC, Squamous Cell Carcinoma; LMICs, Low- and Middle-Income Countries; OS, Overall survival; PORT, Postoperative radiotherapy; RAMED, Régime d’Assistance Médicale (Moroccan medical assistance system); RT, Palliative radiotherapy..

## Conflicts of interest

The authors declare no competing interests.

## Funding

None.

## Ethics approval and consent to participate

The Marrakech Faculty of Medicine and Pharmacy’s Ethical Review Committee waived its approval for the study. It was not necessary to obtain the patient’s informed permission. Before analysis, patient records were anonymised to ensure confidentiality. All methods were performed according to the relevant guidelines and regulations. The need for written informed consent was waived by The Marrakech University Hospital Ethics Committee due to the retrospective nature of the study.

## Consent for publication

Not applicable.

## Availability of data and materials

The datasets used and/or analysfed during the current study are available from the corresponding author on reasonable request.

## Author contributions

**Conception and design:** Anass Baladi, Hassan Abdelilah Tafenzi, Rhizlane Belbaraka.

**Statistical analysis:** Anass Baladi, Hassan Abdelilah Tafenzi.

**Data interpretation:** All authors.

**Financial support:** Anass Baladi, Hassan Abdelilah Tafenzi, Rhizlane Belbaraka.

**Administrative support:** Bioscience and Health Laboratory, Faculty of Medicine and Pharmacy, Cadi Ayyad University, Marrakech, Morocco & Medical Oncology Department, Mohammed VI University Hospital, Marrakech, Morocco.

**Provision of study materials or patients:** Anass Baladi, Hassan Abdelilah Tafenzi, Rhizlane Belbaraka.

**Drafting:** Anass Baladi, Hassan Abdelilah Tafenzi.

**Review, revise and approve the manuscript:** All authors.

## Author information

Anass Baladi (anassbaladi2020@gmail.com), Medical Oncology Resident

Hassan Abdelilah Tafenzi (hassanabdelilah.tafenzi@gmail.com), PhD student

Fatim-Zahra Megzar (megzar.fatim.zahra@gmail.com), Medical Oncology Resident

Ibrahima Kalil Cisse (ibkalilcisse@gmail.com), Medical Oncology Doctor

Othmane Zouiten (drzouitenothmane@gmail.com), Professor of Medical Oncology

Leila Afani (afanileila@gmail.com), Professor of Medical Oncology

Ismail Essaadi (ismail_onco@yahoo.fr), Professor and Head of Medical Oncology department, Avicenna Military Hospital of Marrakech, Morocco

Mohammed El Fadli (elfadli.mohamed2000@gmail.com), Professor of Medical Oncology

Rhizlane Belbaraka (belbaraka.r@gmail.com), Professor and Head of Medical Oncology department, Mohammed VI University Hospital of Marrakech, Morocco.

## Figures and Tables

**Table 1.** Clinical characteristics of ESCC patients by stage at diagnosis (Stage III versus IV) at Mohammed VI Oncological Center, Marrakech (2014–2019).

Variables	Overall, *N* = 78^1^	Stage at diagnosis		*p*-value^2^
		III, *N* = 42^1^	IV, *N* = 36^1^	
Age	56 (48, 66)	56 (46, 64)	60 (50, 68)	0.38
Sex				0.14
Female	46 (59%)	28 (67%)	18 (50%)	
Male	32 (41%)	14 (33%)	18 (50%)	
Tumor location				0.89
Inferior	11 (14%)	5 (12%)	6 (17%)	
Thoracic	41 (53%)	22 (52%)	19 (54%)	
Superior	25 (33%)	15 (36%)	(29%)	
Missing data	1 (1%)	0 (0%)	1(1%)	
Tumor appearance				0.79
Budding	3 (4 %)	2 (5 %)	1 (3 %)	
Infiltrative	7 (9 %)	4 (9 %)	3 (9 %)	
Unspecified	2 (3 %)	0 (0%)	2 (6 %)	
Stenosing	36 (48%)	20 (48%)	16 (48%)	
Ulcerative-budding and stenosing	14 (19%)	9 (21%)	5 (15%)	
Ulcerative + infiltrative	8 (11%)	5 (12%)	3 (9.1%)	
Ulcerative-budding	5 (6.7%)	2 (4.8%)	3 (9.1%)	
Missing data	3 (3.8%)	0 (0%)	3(8.3%)	
ECOG PS				<0.001
1	45 (58%)	30 (73%)	15 (42%)	
2	16 (21%)	10 (24%)	6 (17%)	
3	5 (6.5%)	1 (2.4%)	4 (11%)	
4	11 (14%)	0 (0%)	11 (31%)	
Missing data	1 (1.2%)	1 (1.2%)	0 (0%)	
Treatment modalities				
Chemotherapy	56 (72%)	33 (79%)	23 (64%)	0.15
Neoadjuvant	13 (16.6%)	13 (40%)	N/A	
Concurrent	10 (12.8%)	10 (30%)	N/A	
Palliative	33 (42.3%)	10 (30%)	23 (64%)	
RT	36 (46%)	24 (57%)	12 (33%)	0.035
Localized	24 (30.7%)	24 (57%)	N/A	
Metastatic	12 (15.3%)	N/A	12 (33%)	
Number of sessions (Median)	N/A	15	10	
Total fractions (Median)	N/A	30	20	
Surgery	2 (2.6%)	1 (2.4%)	1 (2.8%)	>0.99
Sites of distant metastases				
Lung metastasis	19 (24%)	N/A	19 (53%)	<0.001
Liver metastasis	7 (9.0%)	N/A	7 (19%)	0.003
Bone metastasis	6 (7.7%)	N/A	6 (17%)	0.008
Adrenal metastasis	2 (2.6%)	N/A	2 (5.6%)	0.21
Tumor stage (T)				0.20
T1	1 (3.0%)	0 (0%)	1 (6.7%)	
T2	2 (6.1%)	0 (0%)	2 (13%)	
T3	4 (12%)	3 (17%)	1 (6.7%)	
T4	26 (79%)	15 (83%)	11 (73%)	
Missing data	45 (57,6%)	24 (57,1%)	21 (58,3%)	
Nodal stage (N)				0.81
N0	18 (53%)	11 (61%)	7 (44%)	
N1	4 (12%)	2 (11%)	2 (12%)	
N2	7 (21%)	3 (17%)	4 (25%)	
N3	5 (15%)	2 (11%)	3 (19%)	
Missing data	44 (57%)	18 (43%)	20 (55%)	
Cancer stage				<0.001
IIIA	20 (26%)	20 (48%)	0 (0%)	
IIIB	15 (19%)	15 (36%)	0 (0%)	
IIIC	7 (9.0%)	7 (17%)	0 (0%)	
IV	36 (46%)	0 (0%)	36 (100%)	
Tobacco use	14 (35%)	8 (38%)	6 (32%)	0.67
Comorbidities				0.80
None	30 (75%)	17 (81%)	13 (68%)	
Cardiac	5 (12%)	2 (9.5%)	3 (16%)	
Endocrine	4 (10%)	2 (9.5%)	2 (11%)	
Pulmonary	1 (2.5%)	0 (0%)	1 (5.3%)	
Symptoms				
Dysphagia	72 (92%)	37 (88%)	35 (97%)	0.21
Vomiting	33 (42%)	16 (38%)	17 (47%)	0.42
IGC	46 (59%)	23 (55%)	23 (64%)	0.41
Epigastric pain	14 (18%)	9 (21%)	5 (14%)	0.39
Dyspnea	3 (3.8%)	1 (2.4%)	2 (5.6%)	0.59
Treatment				0.87
5FU	12 (15%)	7 (17%)	5 (14%)	
Cisplatin	31 (40%)	18 (43%)	13 (36%)	
Docetaxel	2 (2.6%)	1 (2.4%)	1 (2.8%)	
Folfox	1 (1.3%)	1 (2.4%)	0 (0%)	
No treatment	22 (28%)	9 (21%)	13 (36%)	
Xeloda	7 (9.0%)	4 (9.5%)	3 (8.3%)	
Xelox	3 (3.8%)	2 (4.8%)	1 (2.8%)	

^1^Median (IQR); *n* (%)

^2^Wilcoxon rank sum test; Pearson’s chi-squared test; Fisher’s exact test

**Abbreviations:** IGC: Impairment of the General Condition; ESCC: Squamous Cell Carcinoma; ECOG PS: Eastern Cooperative Oncology Group Performance Status; N/A: Not applicable

**Table 2.** Univariate and multivariate cox regression analysis of ESCC patients at Mohammed VI Oncological Center, Marrakech (2014-2019).

	Univariate cox analysis			Multivariate cox aalysis		
Characteristic	HR*^1^*	95% CI*^1^*	*p*-value	HR*^1^*	95% CI*^1^*	*p*-value
Age	1.03	1.00, 1.06	0.022	0.99	0.94, 1.04	0.7
Sex						
Female	—	—				
Male	1.52	0.77, 3.00	0.2			
Siege						
Inferior	—	—				
Thoracic	0.84	0.3, 2.36	0.7			
Superior	0.7	0.24, 2.23	0.6			
PS						
1	—	—		—	—	
2	2.05	0.77, 5.43	0.2	1.96	0.16, 24.7	0.6
3	15.1	4.74, 48.1	<0.001	27.2	1.87, 394	0.015
4	4.83	2.03, 11.4	<0.001	2.38	0.29, 19.8	0.4
CMT						
No	—	—		—	—	
Yes	0.13	0.06, 0.27	<0.001	0.48	0.07, 3.36	0.5
RTH						
No	—	—		—	—	
Yes	0.24	0.10, 0.54	<0.001	0.07	0.01, 0.86	0.038
Lung metastasis						
No	—	—				
Yes	1.73	0.84, 3.53	0.14			
Liver metastasis						
No	—	—				
Yes	0.89	0.31, 2.56	0.8			
Bone metastasis						
No	—	—		—	—	
Yes	4.70	1.73, 12.8	0.002	1.34	0.20, 9.05	0.8
Adrenal metastasis						
No	—	—				
Yes	2.52	0.60, 10.6	0.2			
Nodal stage						
0	—	—				
1	2.97	0.54, 16.2	0.2			
2	2.92	0.88, 9.72	0.080			
3	4.66	1.19, 18.2	0.027			
Metastasis						
0	—	—				
1	2.10	0.82, 5.34	0.12			
Tabac						
No	—	—		—	—	
Yes	4.33	1.57, 12.0	0.005	4.30	0.53, 34.7	0.2
Comorbidities						
No	—	—				
Cardiac	0.44	0.1, 1.98	0.3			
Endocrine	0.74	0.17, 3.29	0.7			
Pulmonary	1.26	0.16, 3.8	0.8			
Cancer stage						
III	—	—		—	—	
IV	5.50	2.36, 12.8	<0.001	2.80	0.50, 15.7	0.2
Dysphagy						
No	—	—				
Yes	1.85	0.44, 7.80	0.4			
Vomitting						
No	—	—				
Yes	0.77	0.39, 1.53	0.5			
AEG						
No	—	—				
Yes	0.88	0.44, 1.75	0.7			
Epigastric						
No	—	—				
Yes	1.09	0.47, 2.52	0.8			
Dyspnea						
No	—	—		—	—	
Yes	4.43	0.99, 19.9	0.052	2.80	0.50, 15.7	0.2

*^1^*HR = Hazard Ratio, CI = Confidence Interval

**Abbreviations:** IGC: Impairment of the General Condition; ESCC: Squamous Cell Carcinoma; ECOG PS: Eastern Cooperative Oncology Group Performance Status; HR: Hazard Ratio; CI: Confidence Interval
